# The plasma peptides of ovarian cancer

**DOI:** 10.1186/s12014-018-9215-z

**Published:** 2018-12-21

**Authors:** Jaimie Dufresne, Pete Bowden, Thanusi Thavarajah, Angelique Florentinus-Mefailoski, Zhuo Zhen Chen, Monika Tucholska, Tenzin Norzin, Margaret Truc Ho, Morla Phan, Nargiz Mohamed, Amir Ravandi, Eric Stanton, Arthur S. Slutsky, Claudia C. dos Santos, Alexander Romaschin, John C. Marshall, Christina Addison, Shawn Malone, Daren Heyland, Philip Scheltens, Joep Killestein, Charlotte E. Teunissen, Eleftherios P. Diamandis, K. W. Michael Siu, John G. Marshall

**Affiliations:** 1 Ryerson Analytical Biochemistry Laboratory (RABL), Department of Chemistry and Biology, Ryerson University, Toronto, Canada; 2 Institute of Cardiovascular Sciences, St Boniface Hospital Research Center, University of Manitoba, Winnipeg, Canada; 3 Division of Cardiology, Department of Medicine, McMaster University, Hamilton, Canada; 4 Keenan Chair in Medicine, St. Michael’s Hospital, University of Toronto, Toronto, Canada; 5 Keenan Research Centre for Biomedical Science, St. Michael’s Hospital, Toronto, Canada; 6 Program for Cancer Therapeutics, Ottawa Hospital Research Institute, Ottawa, Canada; 7 Clinical Evaluation Research Unit, Kingston General Hospital, Kingston, Canada; 8 Alzheimer Center, Department of Neurology, Amsterdam Neuroscience, Amsterdam University Medical Centers, Vrije Universiteit, Amsterdam, The Netherlands; 9 MS Center, Department of Neurology, Amsterdam Neuroscience, Amsterdam University Medical Centers, Vrije Universiteit, Amsterdam, The Netherlands; 10 Neurochemistry Lab and Biobank, Department of Clinical Chemistry, Amsterdam Neuroscience, Amsterdam University Medical Centers, Vrije Universiteit, Amsterdam, The Netherlands; 11 Mount Sinai Hospital Research Institute, University of Toronto, Toronto, Canada; 12 University of Windsor, Windsor, Canada; 13 International Biobank of Luxembourg (IBBL), Luxembourg Institute of Health (formerly CRP Sante Luxembourg), Strassen, Luxembourg; 14 Department of Chemistry and Biology, Faculty of Science, Ryerson University, 350 Victoria St., Toronto, ON Canada

**Keywords:** Human EDTA plasma, Organic extraction, Nano chromatography, Electrospray ionization tandem mass spectrometry, LC–ESI–MS/MS, Linear quadrupole ion trap, Discovery of variation, Ovarian cancer, Random and independent sampling, Chi Square test and ANOVA, SQL SERVER & R

## Abstract

**Background:**

It may be possible to discover new diagnostic or therapeutic peptides or proteins from blood plasma by using liquid chromatography and tandem mass spectrometry to identify, quantify and compare the peptides cleaved ex vivo from different clinical populations. The endogenous tryptic peptides of ovarian cancer plasma were compared to breast cancer and female cancer normal controls, other diseases with their matched or normal controls, plus ice cold plasma to control for pre-analytical variation.

**Methods:**

The endogenous tryptic peptides or tryptic phospho peptides (i.e. without exogenous digestion) were analyzed from 200 μl of EDTA plasma. The plasma peptides were extracted by a step gradient of organic/water with differential centrifugation, dried, and collected over C18 for analytical HPLC nano electrospray ionization and tandem mass spectrometry (LC–ESI–MS/MS) with a linear quadrupole ion trap. The endogenous peptides of ovarian cancer were compared to multiple disease and normal samples from different institutions alongside ice cold controls. Peptides were randomly and independently sampled by LC–ESI–MS/MS. Precursor ions from peptides > E4 counts were identified by the SEQUEST and X!TANDEM algorithms, filtered in SQL Server, before testing of frequency counts by Chi Square (χ^2^), for analysis with the STRING algorithm, and comparison of precursor intensity by ANOVA in the R statistical system with the Tukey-Kramer Honestly Significant Difference (HSD) test.

**Results:**

Peptides and/or phosphopeptides of common plasma proteins such as HPR, HP, HPX, and SERPINA1 showed increased observation frequency and/or precursor intensity in ovarian cancer. Many cellular proteins showed large changes in frequency by Chi Square (χ^2^ > 60, *p* < 0.0001) in the ovarian cancer samples such as ZNF91, ZNF254, F13A1, LOC102723511, ZNF253, QSER1, P4HA1, GPC6, LMNB2, PYGB, NBR1, CCNI2, LOC101930455, TRPM5, IGSF1, ITGB1, CHD6, SIRT1, NEFM, SKOR2, SUPT20HL1, PLCE1, CCDC148, CPSF3, MORN3, NMI, XTP11, LOC101927572, SMC5, SEMA6B, LOXL3, SEZ6L2, and DHCR24. The protein gene symbols with large Chi Square values were significantly enriched in proteins that showed a complex set of previously established functional and structural relationships by STRING analysis. Analysis of the frequently observed proteins by ANOVA confirmed increases in mean precursor intensity in ZFN91, TRPM5, SIRT1, CHD6, RIMS1, LOC101930455 (XP_005275896), CCDC37 and GIMAP4 between ovarian cancer versus normal female and other diseases or controls by the Tukey–Kramer HSD test.

**Conclusion:**

Here we show that separation of endogenous peptides with a step gradient of organic/water and differential centrifugation followed by random and independent sampling by LC–ESI–MS/MS with analysis of peptide frequency and intensity by SQL Server and R revealed significant difference in the ex vivo cleavage of peptides between ovarian cancer and other clinical treatments. There was striking agreement between the proteins discovered from cancer plasma versus previous biomarkers discovered in tumors by genetic or biochemical methods. The results indicate that variation in plasma proteins from ovarian cancer may be directly discovered by LC–ESI–MS/MS that will be a powerful tool for clinical research.

**Electronic supplementary material:**

The online version of this article (10.1186/s12014-018-9215-z) contains supplementary material, which is available to authorized users.

## Introduction

### Blood peptides

Blood peptides may be identified by C18 liquid chromatography electrospray ionization and tandem mass spectrometry (LC-ESI-MS/MS) [1]. The endogenous peptides of human blood were first identified by MS/MS fragmentation that demonstrated that a tryptic like endoproteinase activity cleaves peptides from proteins but an exopeptidase activity degraded the peptides creating a pseudo steady state [1–5]. The alternative RNA splicing of pre, pro or protein substrates combined with complex pathways of post translational processing may result in the cleavage of many peptides from proteins in circulation that may help mediate, or mark, important physiological processes [6]. Protein cleavage products from pro-opiomelanocortin, natriuretic peptides, insulin like growth factors, coagulation factor XIII, proglucagon-derived peptides, human kallikrein-related peptidase SERPINA1, ENOSF1, neurofilament medium polypeptide, circulating IGFBP-4 fragments and many others have been suggested to have some diagnostic or mechanistic importance [7–18]. Multivariate analysis provided about the same statistical power compared to univariate ANOVA of the main feature(s) [1, 19, 20]. Random and independent sampling of the endogenous tryptic peptides from clinical plasma samples revealed individual analytes that show significant variation by standard statistical tests such as the Chi Square test and ANOVA [1, 2, 4, 21–23]. Pre-analytical variation was exhaustively studied between fresh EDTA plasma samples on ice versus plasma samples degraded for various lengths of time to control for differences in sample handling and storage and showed the observation of peptides from many proteins may increase by on average twofold after incubation at room temperature [2–4] but that Complement C3 and C4B vary sharply with incubation time [2, 4] in agreement with previous results [1].

### Sample preparation

Without pre-fractionation, only peptides from a few high abundance proteins may be observed by LC-ESI-MS/MS [24–26]. In contrast, with one step sample preparation by partition chromatography or differential centrifugation, low abundance proteins of ~ 1 ng/ml could be detected and quantified in blood samples by electrospray mass spectrometry [26–28]. The sensitive analysis of human blood fluids by LC–ESI–MS/MS is dependent on selective fractionation strategies, such as partition chromatography or organic extraction, to relieve suppression and competition for ionization, resulting in high signal to noise ratios and thus low error rates of identification and quantification [28]. Simple and single-use, i.e. disposable, preparative and analytical separation apparatus permits the identification and quantification of blood peptides and proteins with no possibility of cross contamination between patients that guarantees sampling is statistically independent [1, 2, 25–27]. Previously, the use of precipitation and selective extraction of the pellet [5, 27, 29, 30] was shown to be superior to precipitation and analysis of the ACN supernatant [31], ultra-filtration, [32] albumin depletion chromatography [33] or C18 partition chromatography alone [25]. Precipitating all of the polypeptides with 90% ACN followed by step-wise differential centrifugation with mixtures of organic solvent and water was the optimal method to sensitively detect endogenous peptides from cellular proteins in blood [24]. Here a ten-step gradient of acetonitrile/water with differential centrifugation to extract 200 µl of EDTA plasma for analysis by LC–ESI–MS/MS showed a high signal to noise ratio [24] and resulted in the confident identification of tryptic peptides [2] from ovarian cancer versus normal control samples.

### Computation

Partitioning each clinical sample into multiple selective sub-fractions, that each must be separately resolved by analytical C18, provides sensitivity [24] but creates a computational challenge. Previously the 32 bit computer power was lacking to compare all the peptides of all the proteins of the many sub-factions from each patient in a large experiment [34]. At present the MS/MS spectra from random and independent sampling of peptides from thousands of LC–ESI–MS/MS may be fit to peptides using a 64 bit server and then compared across treatments using SQL SERVER/R that provides excellent data compression, relation and analysis [2, 21]. The protein p-values and FDR q-values as well as the peptide-to-protein distribution of the precursor ions of > 10,000 counts from organic extraction were confirmed against a null (i.e. known false positive) model of noise or computer generated random MS/MS spectra [2, 22, 35–37]. The standard SQL Server system permits the direct interrogation of the related data by the open source R statistical system without proteomic-specific software packages. Here for the first time the use of SQL/R has permitted the detailed statistical analysis of randomly and independently sampled LC–ESI–MS/MS data from multiple clinical locations and treatments in parallel that would be requisite for a multisite clinical trial.

### Cancer proteins in blood fluids

Many non-specific, i.e. common, or so called “acute phase” proteins have been detected to increase by the analysis of blood fluids such as amyloids, complement, haptoglobin, alpha 1 antitrypsin, clusterin, (ApoJ), complement components, heat shock proteins, fibrinogens, hemopexin, alpha 2 macroglobulin and others that may be of limited diagnostic value [28, 38, 39]. There is good evidence that cellular proteins may exist in circulation, and even form supramolecular complexes with other molecules in the blood [40]. Proteins and RNA may be packaged in exosomes [41, 42] that are challenging to isolate and it appears that supramolecular complexes of proteins, including DNA/RNA binding proteins, from cells may exist in circulation [40, 43, 44]. Apolipoprotein A IV (APOA4) and vitamin D binding protein (VDBP) significantly discriminated malignant from benign cases of ovarian cancer but was not as good as CA125 for diagnostic accuracy [45]. A proteomic signature of ovarian cancer tumor fluid was identified and verified by targeted proteomics [46]. Protein Z was identified as a putative novel biomarker for early detection of ovarian cancer [47]. Cystatin B (CYTB) may be a potential diagnostic biomarker in ovarian clear cell carcinoma [48]. Here, the combination of step wise organic partition [24], random and independent sampling by nano electrospray LC–ESI–MS/MS, and large scale 64 bit computation with SQL SERVER/R [21] permitted the sensitive detection of peptides and/or phosphopeptides, and thus the presence of the parent protein chains and complexes, from human plasma for comparison of variation in ovarian cancer patients versus controls by the classical statistical approaches of the Chi Square test followed by univariate ANOVA [1, 22, 23].

## Materials and methods

### Materials

The HPLC was an Agilent 1100 (Santa Clara CA USA). The linear ion trap mass spectrometer was an LTQ XL (Thermo Electron Corporation, Waltham, MA, USA). The anonymous human EDTA plasma (9–20 per disease or normal control) with no identifying information was obtained from multiple clinical locations of St Joseph’s Hospital of McMaster University, The Ontario Tumor Bank of the Ontario Institute of Cancer Research, St Michaels Hospital Toronto, Amsterdam University Medical Centers, Vrije Universiteit Amsterdam, and IBBL Luxembourg under Ryerson Ethic Review Board Protocol REB 2015-207. The arbitrarily selected disease population samples were from patients that received a confirmed diagnoses of the disease indicated at the source institution. The plasma samples were collected before therapeutic intervention and no additional information about the samples were made available. C18 ZipTips were obtained from Millipore (Bedford, MA). C18 HPLC resin was from Agilent (Zorbax 300 SB-C18 5-micron). Solvents were obtained from Caledon Laboratories (Georgetown, Ontario, Canada). All other salts and reagents were obtained from Sigma-Aldrich-Fluka (St Louis, MO) except where indicated.

### Sample preparation

Human EDTA plasma samples (200 μl) were precipitated with 9 volumes of acetonitrile (90% ACN) [27], followed by the selective extraction of the pellet using a step gradient to achieve selectivity across sub-fractions and thus greater sensitivity [24]. Disposable plastic 2 ml sample tubes and plastic pipette tips were used to handle samples. The acetonitrile suspension was separated with a centrifuge at 14,000 RCF for 5 min. The acetonitrile supernatant, that contains few peptides, was collected, transferred to a fresh sample tube and dried in a rotary lyophilizer. The organic precipitate (pellet) that contains a much larger total amount of endogenous polypeptides [27] was manually re-suspended using a step gradient of increasing water content to yield 10 fractions from those soluble in 90% ACN to 10% ACN, followed by 100% H_2_O, and then 5% formic acid [24]. The extracts were clarified with a centrifuge at 14,000 RCF for 5 min. The extracted sample fractions were dried under vacuum in a rotary lyophilizer and stored at − 80 °C for subsequent analysis.

### Preparative C18 chromatography

The peptides of EDTA plasma precipitated in ACN, and extracted from the pellet in a step-gradient were then re-dissolved in 5% formic acid and collected over C18 preparative partition chromatography. Preparative C18 separation provided the best results for peptide and phosphopeptide analysis in a “blind” analysis [49]. Solid phase extraction with C18 for LC–ESI–MS/MS was performed as previously described [1, 25–27, 29]. The C18 chromatography resin (Zip Tip) was wet with 65% acetonitrile before equilibration in water with 5% formic acid. The plasma extract was dissolved in 200 μl of 5% formic acid in water. The resin was washed with at least five volumes of the same binding buffer. The resin was eluted with ≥ 3 column volumes of 65% acetonitrile (2 µl) in 5% formic acid. In order to avoid cross-contamination the preparative C18 resin was discarded after a single use.

### LC–ESI–MS/MS

In order to entirely prevent any possibility of cross contamination, a new disposable nano analytical HPLC column and nano emitter was fabricated for recording each patient sample-fraction set. The ion traps were cleaned and tested for sensitivity with angiotensin and glu-fibrinogen prior to recordings. The new column was conditioned and quality controlled with a mixture of three non-human protein standards using a digest of Bovine Cytochrome C, Yeast alcohol dehydrogenase (ADH) and Glycogen Phosphorylase B to confirm the sensitivity and mass accuracy of the system prior to each patient sample set [35]. The statistical validity of the linear quadrupole ion trap for LC–ESI–MS/MS of human plasma [24] was in agreement with the results from the 3D Paul ion trap [22, 35–37]. The stepwise extractions were collected and desalted over C18 preparative micro columns, eluted in 2 µl of 65% ACN and 5% formic acid, diluted tenfold with 5% formic acid in water and 5% ACN, and immediately loaded manually into a 20 μl metal sample loop before injecting onto the analytical column via a Rhodynne injector. Endogenous peptide samples were analyzed over a discontinuous gradient generated at a flow rate of ~ 10 μl per minute with an Agilent 1100 series capillary pump split upstream of the injector during recording to about ~ 200 nl per minute. The separation was performed with a C18 (150 mm × 0.15 mm) fritted capillary column. The acetonitrile profile was started at 5%, ramped to 12% after 5 min and then increased to 65% over ~ 90 min, remained at 65% for 5 min, decreased to 50% for 15 min and then declined to a final proportion of 5% prior to injection of the next step fraction from the same patient. The nano HPLC effluent was analyzed by ESI ionization with detection by MS and fragmentation by MS/MS with a linear quadrupole ion trap [50]. The instrument was set to collect the precursor for up to 200 ms prior to MS/MS fragmentation with up to four fragmentations per precursor ion that were averaged. Individual, independent samples from disease, normal and ice cold control were precipitated, fractionated over a step gradient and collected over C18 for manual injection.

### Correlation analysis

In this study we accepted about 15 million precursor ions with intensity > E4 counts that was previously shown to be at the 99% percentile of the noise distribution with an average signal to noise of approximately one hundred [2, 24]. Correlation analysis of ion trap data was performed with the X!TANDEM [51] and SEQUEST [52] algorithms to match tandem mass spectra to peptide sequences from a library of 158,071 unique Homo sapien proteins that differ by at least one amino acid from RIKEN, IMAGE, RefSeq, NCBI, Swiss Prot, TrEMBLE, ENSEMBL, UNIPROT and UNIPARC along with available Gene Symbols, all previous accession numbers, description fields and any other available annotation rendered non-redundant by protein sequence in SQL Server last assembled in May 2015. Endogenous peptides with precursors > 10,000 (E4) arbitrary counts were searched as fully tryptic peptides and/or phosphopeptides and the results compared in SQL Server/R. The X!TANDEM default ion trap data settings of ± 3 *m*/*z* from precursors peptides considered from 300 to 2000 *m*/*z* with a tolerance of 0.5 Da error in the fragments were used [22, 26, 36, 37, 51, 53]. The best fit peptide of the MS/MS spectra to fully tryptic and/or phospho-tryptic peptides at charge states of + 2 versus + 3 were accepted with additional acetylation, or oxidation of methionine and with possible loss of water or ammonia. The resulting accession numbers, actual and estimated masses, correlated peptide sequences, peptide and protein scores, resulting protein sequences and other associated data were captured and assembled together in an SQL Server relational database [21].

### Data sampling, sorting, transformation and visualization

The linear quadrupole ion trap provided the precursor ion intensity and m/z values plus the peptide fragment MS/MS spectra. The MS/MS spectra were redundantly correlated to specific tryptic peptide sequences by the X!TANDEM and SEQUEST algorithms. The MS and MS/MS spectra together with the results of the X!TANDEM and SEQUEST algorithms were parsed into an SQL Server database and filtered [21] before statistical and graphical analysis with the generic R data system [21–23, 35, 54]. The peptide to protein correlation frequency counts for each gene symbol were summed over ovarian cancer versus control to correct the observation frequency prior to the Chi Square test using Eq. (1):

1 $$({\text{i}})\quad \chi2=({\text{Disease}}{-} {\text{Control}})^{2} /({\text{Control}} + 1)$$

The precursor intensity data for MS/MS spectra were log_10_ transformed, tested for normality and analyzed across institution/study and diseases verses controls by means, standard errors, quantile box plots and ANOVA [22, 23, 35]. The Chi Square test, and entirely independent analysis of the precursor intensity using the rigorous ANOVA with Tukey–Kramer HSD test, versus multiple controls was achieved using a 64 bit R server.

## Results

The aim and objective of this study was proof of concept towards a method to compare the endogenous tryptic peptides of ovarian cancer plasma to that from multiple clinical locations that utilized random and independent sampling with a battery of robust and sensitive linear quadrupole ion trap ion traps where the results were compiled using a central SQL Server R statistical system. The method shows great sensitivity and flexibility but relies on the fit of MS/MS spectra to assign peptide identity, and statistical analysis of peptide observation frequency and intensity, and so is computationally intensive.

### LC–ESI–MS/MS

The pool of endogenous tryptic peptides (TRYP) and/or tryptic phosphopeptides (STYP) were randomly and independently sampled without replacement by liquid chromatography, nano electrospray ionization and tandem mass spectrometry (LC–ESI–MS/MS) [2] from ovarian versus breast cancer, or female normal, other disease and normal plasma, and ice cold controls (see Additional file 1: Table S1) to serve as a baseline. The raw correlations were filtered to retain only the best fit by charge state and peptide sequence in SQL Server to entirely avoid re-use of the same MS/MS spectra. The filtered results were then analyzed by the generic R statistical system in a matrix of disease and controls that revealed the set of blood peptides specific to each disease state. The statistical validity of the extraction and sampling system were previously established by computation of cumulative p-values and FDR corrected q-values for each gene symbol by the method of Benjamini and Hochberg [55] and frequency comparison to null (i.e. known false positive) noise or random MS/MS spectra [2, 24]. The experimental LC–ESI–MS/MS resulted in 15,968,550 MS/MS spectra of which 1,916,672 (12%) were fit by X!TANDEM to distinct best fit peptides with p-values that were computed together to provide the cumulative p-value for each protein accession that resulted in over 14,000 types of protein gene symbols with p-values and FDR corrected q-values of < 1/10,000 (q ≤ 0.0001).

### Frequency correction

**Fig. 1:**
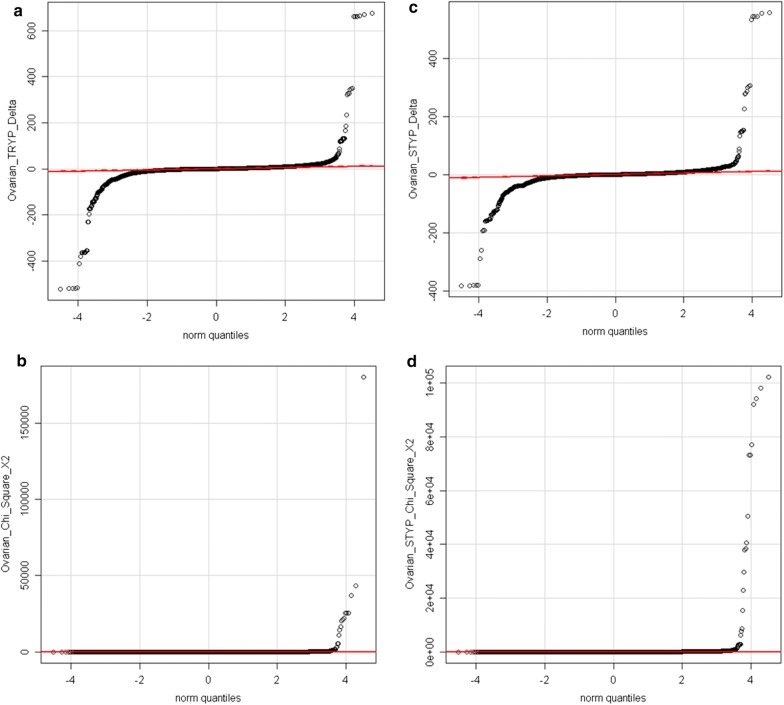
Quantile plots of the corrected difference in observation frequency (Delta) and Chi Square values of the ovarian cancer (i.e. disease treatment) versus control as indicated. The difference of ovarian cancer (n ≥ 10) versus each of the female normal (n ≥ 5) using the Quantile plot tended to zero (see red line). Similar results were obtained by comparison to breast cancer or other controls (not shown). **a** Tryptic peptide corrected difference (delta) in observation frequency; **b** tryptic peptide Chi Square χ^2^; **c** tryptic and/or STYP the corrected difference (delta) in observation frequency; **d** tryptic and/or STYP peptide Chi Square χ^2^

A total of 269,371 tryptic (TRYP) and 274,356 phospho-tryptic (TRYP-STYP) MS/MS were correlated to proteins from female normal plasma. Similarly, 660,251 (TRYP) and 667, 467 (TRYP-STYP) MS/MS were correlated to proteins from ovarian cancer plasma and these sums were used to correct observation frequency. The observed frequency difference plot passed through the 0 point (no difference in observed frequency) at the 0 quantile point (mean of difference distribution) clearly indicating the observation frequency values were proportionally corrected prior to Chi Square comparison (Fig. 1).

### Comparison of ovarian cancer to female normal by Chi Square analysis

A set of ~ 500 gene symbols showed Chi Square (χ^2^) values of ≥ 15 between the ovarian cancer versus the normal female samples. Ovarian-cancer-specific peptides and/or phosphopeptides from cellular proteins, membrane proteins, nucleic acid binding proteins, signaling factors, metabolic enzymes and others including uncharacterized proteins showed significantly greater observation frequency. In agreement with the literature, peptides from many common proteins including acute phase response proteins such as Haptoglobin (HP) [39], Haptoglobin Related Protein (HPR), Alpha Anti Trypsin (SERPINA1) [15] and others were more frequently observed in ovarian cancer samples [38] (Table 1). The Chi Square analysis showed some proteins with χ^2^ values that were apparently far too large (χ^2^ ≥ 60, *p* < 0.0001, *df* 1) to all have resulted from random sampling error (Fig. 1). Many proteins showed an observation frequency that was significantly greater in ovarian cancer plasma including ZNF91, ZNF254, F13A1, LOC102723511, ZNF253, QSER1, P4HA1, GPC6, LMNB2, PYGB, NBR1, CCNI2, LOC101930455, TRPM5, IGSF1, ITGB1, CHD6, SIRT1, NEFM, SKOR2, SUPT20HL1, PLCE1, CCDC148, CPSF3, MORN3, NMI, XTP11, LOC101927572, SMC5, SEMA6B, LOXL3, SEZ6L2 and DHCR24 (Table 1). The full list of Chi Square results are found in Additional file 2: Table S2.

**Table 1:** Ovarian cancer specific proteins detected by fully tryptic peptides and/or fully tryptic phosphopeptides that show a Chi Square (χ^2^) value of ≥ 60

Gene symbol	Average X2 statistic per gene symbol	Accessions per gene symbol
*Fully tryptic peptides*		
HP	2.61E+04	14
HPR	7.27E+03	3
SERPINA1	2.42E+03	6
ZNF91	1.52E+03	7
ZNF254	5.29E+02	9
C4B_2	4.89E+02	1
HPX	4.33E+02	1
F13A1	3.13E+02	8
LOC102723511	3.10E+02	1
ZNF253	3.03E+02	2
QSER1	2.92E+02	6
P4HA1	2.59E+02	3
GPC6	2.53E+02	2
LMNB2	2.22E+02	2
PYGB	1.73E+02	2
C4A	1.64E+02	6
NBR1	1.61E+02	11
CCNI2	1.60E+02	3
LOC101930455	1.39E+02	1
TRPM5	1.38E+02	6
IGSF1	1.26E+02	6
ALB	1.18E+02	8
ITGB1	1.08E+02	15
CHD6	1.07E+02	8
SIRT1	1.04E+02	5
NEFM	1.02E+02	5
SKOR2	1.00E+02	3
C4B	9.94E+01	10
SUPT20HL1	9.93E+01	2
PLCE1	9.83E+01	8
CFB	9.65E+01	7
SRGN	8.87E+01	1
DGCR14	8.69E+01	5
SOWAHC	8.64E+01	1
DKFZp434P0729	8.64E+01	1
HEL-S-82p	8.64E+01	1
USP45	8.35E+01	16
ST8SIA2	7.87E+01	5
REST	7.80E+01	17
ANKRD49	7.51E+01	6
GPR101	7.49E+01	1
TMC3	7.46E+01	2
TAT	6.96E+01	2
*Phosphotryptic peptides*		
HP	4.35E+04	14
HPR	2.62E+04	3
SERPINA1	2.56E+03	6
CCDC148	2.17E+03	11
CPSF3	1.26E+03	5
MORN3	1.06E+03	1
C4B_2	8.88E+02	1
QSER1	6.28E+02	6
SIRT1	6.26E+02	5
CCNI2	4.62E+02	3
NMI	3.45E+02	3
Nbla03646	3.45E+02	1
XTP11	3.45E+02	1
HPX	3.35E+02	1
LOC101927572	3.28E+02	1
F13A1	3.22E+02	8
SMC5	3.11E+02	4
C4A	3.01E+02	6
SEMA6B	2.85E+02	2
LOXL3	2.81E+02	10
SEZ6L2	2.31E+02	9
DHCR24	2.24E+02	4
RTTN	2.23E+02	8
DBR1	2.18E+02	3
ALCAM	2.08E+02	6
LOC401437	2.03E+02	1
BAI1	2.02E+02	3
NID2	1.92E+02	8
SOWAHC	1.91E+02	1
C6orf165	1.90E+02	3
C4B	1.80E+02	10
FGA	1.75E+02	6
RGS22	1.75E+02	15
OXER1	1.69E+02	2
ARHGEF25	1.60E+02	3
hCG_2031321	1.60E+02	1
FAM110B	1.58E+02	1
LOC102725271	1.58E+02	1
ORC1	1.58E+02	2
ORC1L	1.58E+02	1
VWA5B1	1.57E+02	10
KCNQ2	1.57E+02	15
DGKH	1.54E+02	5
PTGFRN	1.53E+02	4
CCDC37	1.52E+02	3
DKFZp686H14204	1.48E+02	1
ISL1	1.47E+02	2
GIMAP4	1.45E+02	4
LOC375295	1.44E+02	1

### Pathway and gene ontology analysis using the STRING algorithm

**Fig. 2:**
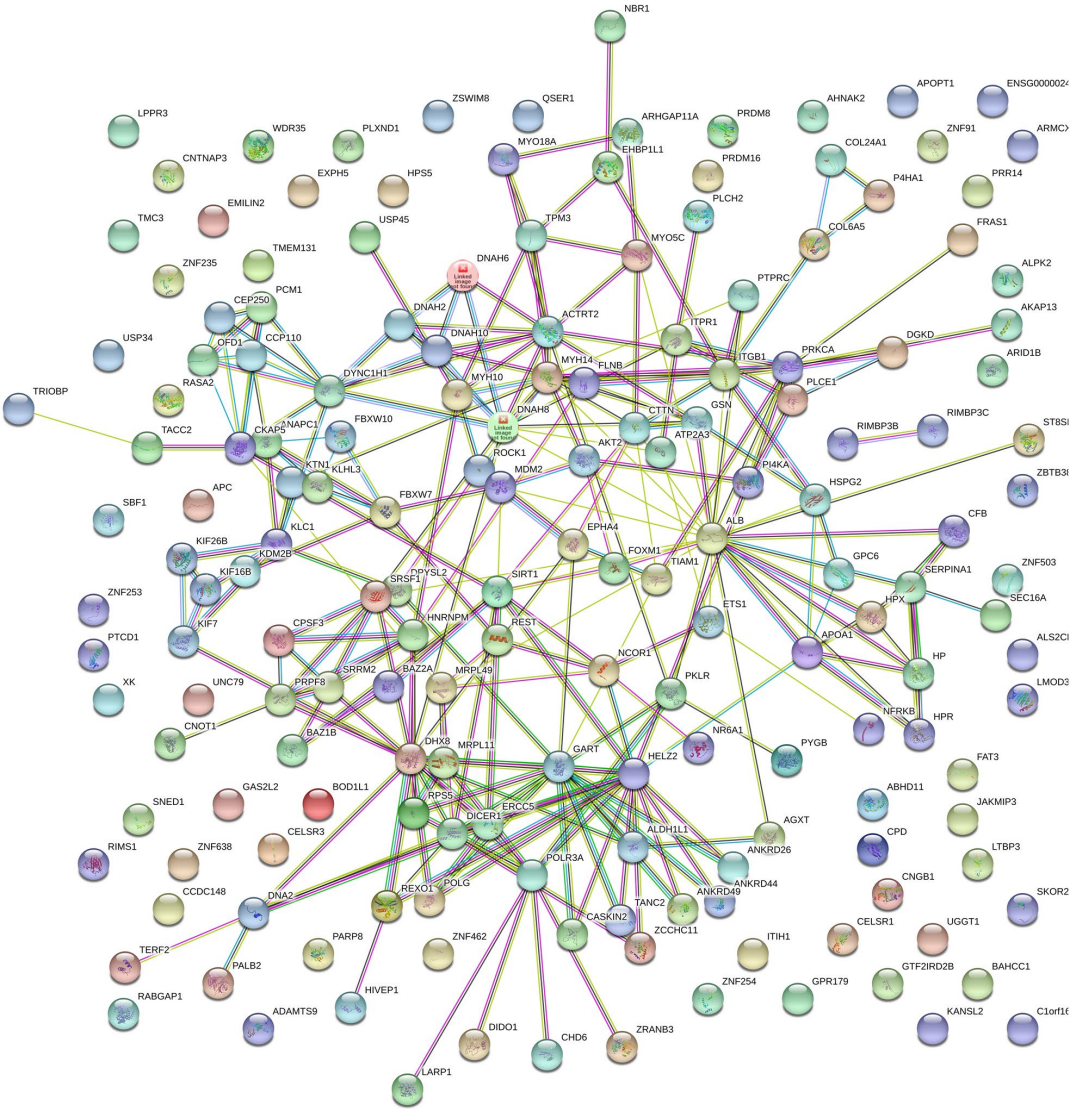
The Ovarian Cancer STRING network where Chi Square χ^2^ ≥ 15 from fully tryptic peptides. Ovarian Cancer tryptic peptide frequency difference > 15 and χ^2^ value > 15 at degrees of freedom of 1 (*p* < 0.0001). Network Stats: number of nodes, 173; number of edges, 260; average node degree, 3.01; avg. local clustering coefficient, 0.378; expected number of edges, 206; PPI enrichment p-value, 0.000175

**Fig. 3:**
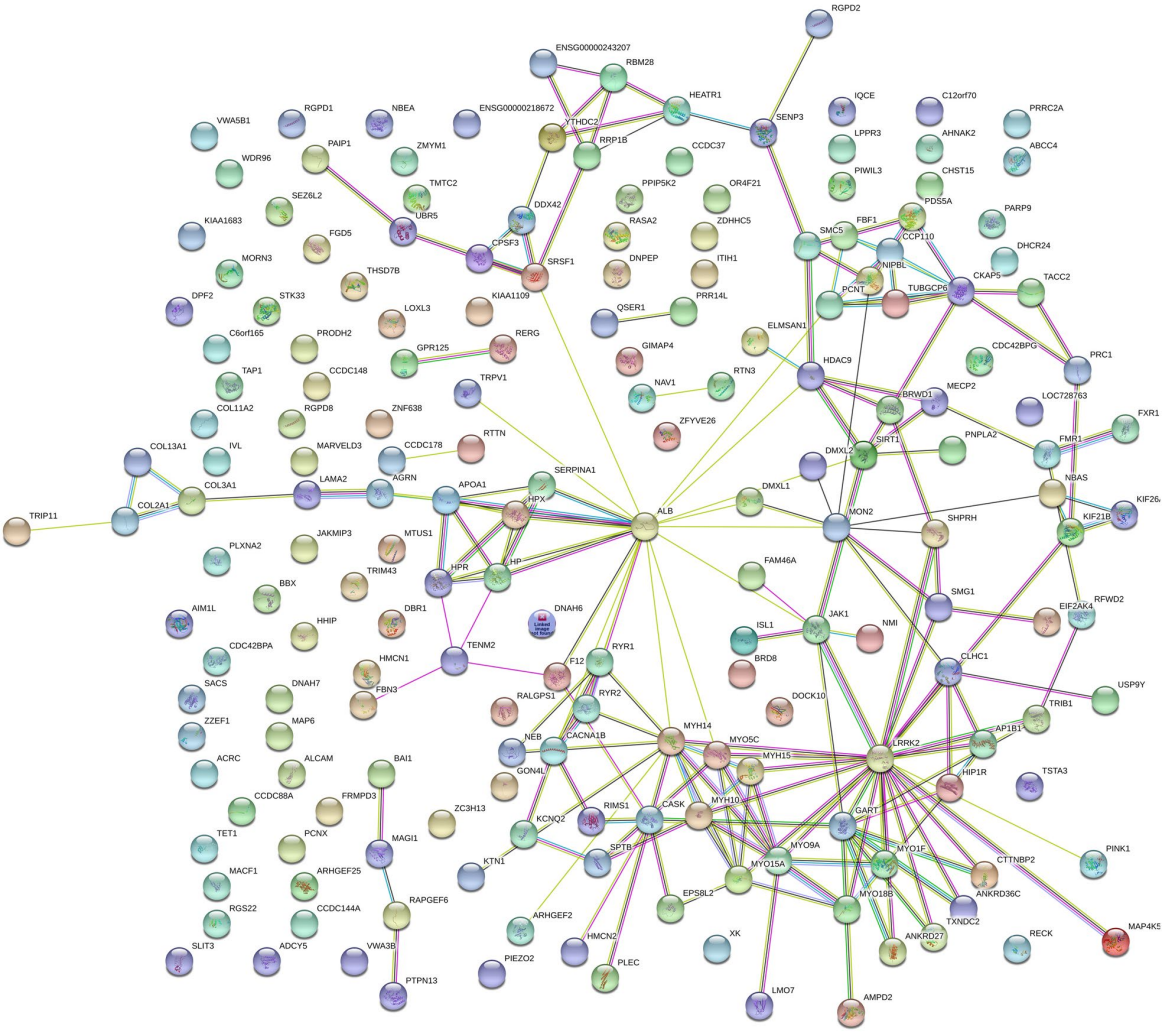
The Ovarian Cancer STRING network where Chi Square χ^2^ ≥ 15 from fully tryptic phospho peptides. Ovarian Cancer STYP, frequency difference > 15 and χ^2^ value > 15 at degrees of freedom of 1 (*p* < 0.0001). Network Information: number of nodes, 191; number of edges, 182; average node degree, 1.91; avg. local clustering coefficient, 0.335; expected number of edges, 152; PPI enrichment p-value, 0.00911

In a computationally independent method to ensure the variation in proteins associated with ovarian cancer were not just the result of some random process, we analyzed the distribution of the known protein–protein interactions and the distribution of the cellular location, molecular function and biological processes of the proteins identified with respect to a random sampling of the human genome. There were many interactions apparent between the proteins computed to be specific to ovarian cancer from fully tryptic (Fig. 2) and/or phospho tryptic peptides (Fig. 3). The ovarian cancer samples showed statistically significant enrichment of protein interactions and Gene Ontology terms that were consistent with structural and functional relationships between the proteins identified in ovarian cancer compared to a random sampling of the human genome (Table 2).

**Table 2:** The summary of STRING analysis with respect to a random sampling of the human genome for gene symbols that show a Chi Square (χ^2^) value ≥ 15 (see Additional file 1: Table S1, Additional file 2: Table S2)

Pathway ID	Pathway description	Count in gene set	False discovery rate
*Biological process (GO)*			
GO:0007017	Microtubule-based process	17	0.00251
GO:0007018	Microtubule-based movement	11	0.00251
GO:1902589	Single-organism organelle organization	34	0.0265
*Molecular function (GO)*			
GO:0003774	Motor activity	12	3.17E−06
GO:0003777	Microtubule motor activity	9	2.55E−05
GO:0043167	Ion binding	81	0.000114
GO:0032559	Adenyl ribonucleotide binding	30	0.00325
GO:0097159	Organic cyclic compound binding	71	0.00325
GO:1901363	Heterocyclic compound binding	70	0.00325
GO:0005524	ATP binding	29	0.004
GO:0036094	Small molecule binding	41	0.00734
GO:0005515	Protein binding	60	0.00741
GO:0005488	Binding	107	0.0114
GO:0043169	Cation binding	56	0.0114
GO:0016887	ATPase activity	12	0.0122
GO:0046872	Metal ion binding	55	0.0122
GO:0043168	Anion binding	40	0.014
GO:0032549	Ribonucleoside binding	30	0.0393
GO:0000166	Nucleotide binding	35	0.0419
*Cellular component (GO)*			
GO:0005875	Microtubule associated complex	10	0.000239
GO:0072562	Blood microparticle	9	0.000239
GO:0032991	Macromolecular complex	62	0.00102
GO:0015630	Microtubule cytoskeleton	23	0.00182
GO:0043233	Organelle lumen	57	0.00182
GO:0044446	Intracellular organelle part	86	0.00317
GO:0044430	Cytoskeletal part	26	0.00399
GO:0030286	Dynein complex	5	0.0049
GO:0044422	Organelle part	86	0.00587
GO:0030426	Growth cone	7	0.00767
GO:0043232	Intracellular non-membrane-bounded organelle	48	0.00767
GO:0070013	Intracellular organelle lumen	53	0.00771
GO:0005868	Cytoplasmic dynein complex	4	0.0102
GO:0005858	Axonemal dynein complex	3	0.0174
GO:0043226	Organelle	116	0.0174
GO:0043234	Protein complex	50	0.0174
GO:0097513	Myosin II filament	2	0.0174
GO:0043229	Intracellular organelle	109	0.0203
GO:0005856	Cytoskeleton	29	0.0272
GO:0030027	Lamellipodium	7	0.0272
GO:0031988	Membrane-bounded vesicle	45	0.0272
GO:0033553	rDNA heterochromatin	2	0.0272
GO:0071682	Endocytic vesicle lumen	3	0.0272
GO:0071013	Catalytic step 2 spliceosome	5	0.0299
GO:0001725	Stress fiber	4	0.0301
GO:0044441	Ciliary part	9	0.0315
GO:0070062	Extracellular exosome	38	0.0318
GO:0005929	Cilium	11	0.0325
GO:0060205	Cytoplasmic membrane-bounded vesicle lumen	5	0.0325
GO:0005874	Microtubule	10	0.0331
GO:0005654	Nucleoplasm	38	0.0343
GO:0005871	Kinesin complex	4	0.0353
GO:0042641	Actomyosin	4	0.0353
GO:0043227	Membrane-bounded organelle	109	0.0363
GO:0042995	Cell projection	25	0.0441
GO:0044463	Cell projection part	16	0.0441
*KEGG pathways*			
5205	Proteoglycans in cancer	11	0.000747
*Molecular function (GO)*			
GO:0003774	Motor activity	12	9.83E−06
GO:0032559	Adenyl ribonucleotide binding	34	0.000836
GO:0005524	ATP binding	33	0.000971
GO:0000166	Nucleotide binding	42	0.00237
GO:0032550	Purine ribonucleoside binding	36	0.00237
GO:0032555	Purine ribonucleotide binding	36	0.00237
GO:0036094	Small molecule binding	45	0.00237
GO:0043168	Anion binding	46	2.37E−03
GO:0035639	Purine ribonucleoside triphosphate binding	35	3.07E−03
GO:0097367	Carbohydrate derivative binding	39	0.00529
GO:0043167	Ion binding	78	0.0171
GO:0031267	Small gtpase binding	8	0.0221
GO:0008092	Cytoskeletal protein binding	14	0.0392
GO:0017111	Nucleoside-triphosphatase activity	17	0.0417
GO:0005219	Ryanodine-sensitive calcium-release channel activity	2	0.0462
*Cellular component (GO)*			
GO:0016459	Myosin complex	8	0.000231
GO:0005737	Cytoplasm	114	0.00491
GO:0005856	Cytoskeleton	35	0.00491
GO:0042995	Cell projection	32	0.00491
GO:0043232	Intracellular non-membrane-bounded organelle	54	0.00491
GO:0016461	Unconventional myosin complex	3	0.00638
GO:0072562	Blood microparticle	7	0.0127
GO:0005874	Microtubule	12	0.0176
GO:0030016	Myofibril	9	0.0176
GO:0044430	Cytoskeletal part	25	0.0315
GO:0097458	Neuron part	22	0.0315
GO:0097513	Myosin II filament	2	0.0315
GO:0044449	Contractile fiber part	8	0.0449
GO:0015630	Microtubule cytoskeleton	20	0.0462
GO:0044463	Cell projection part	18	0.0462
GO:0071682	Endocytic vesicle lumen	3	0.0462
GO:0043005	Neuron projection	18	0.0472

### ANOVA analysis across disease, normal and control plasma treatments

**Fig. 4:**
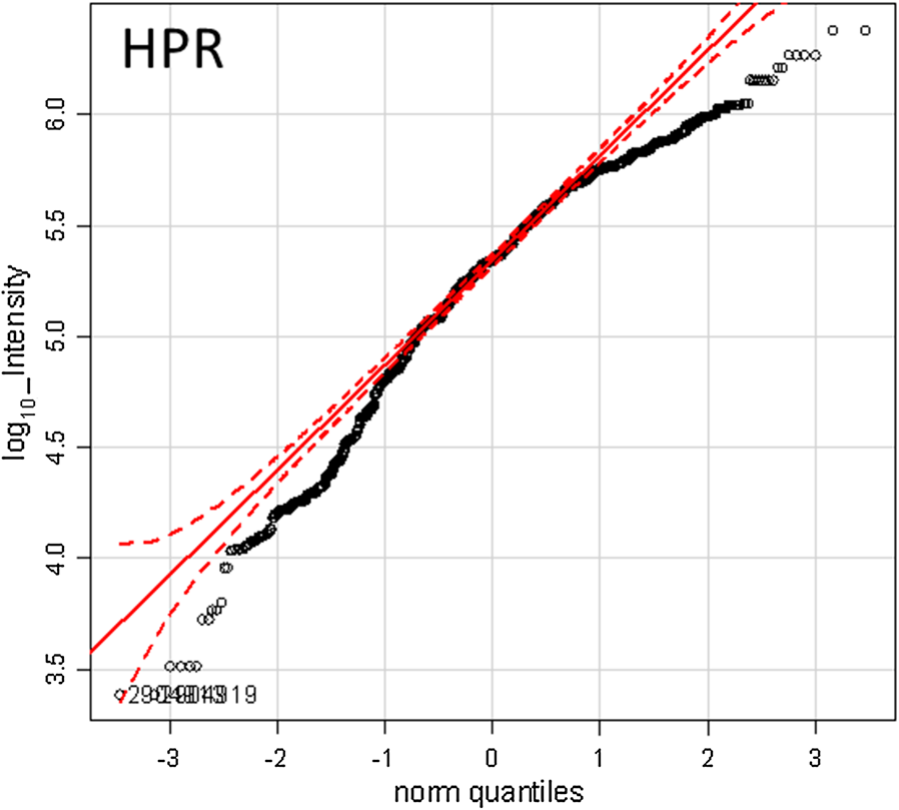
The quantile plot showing the normality of the Log_10_ peptide intensity values of HPR. The dashed red lines define an ideal Gaussian or Normal distribution

**Fig. 5:**
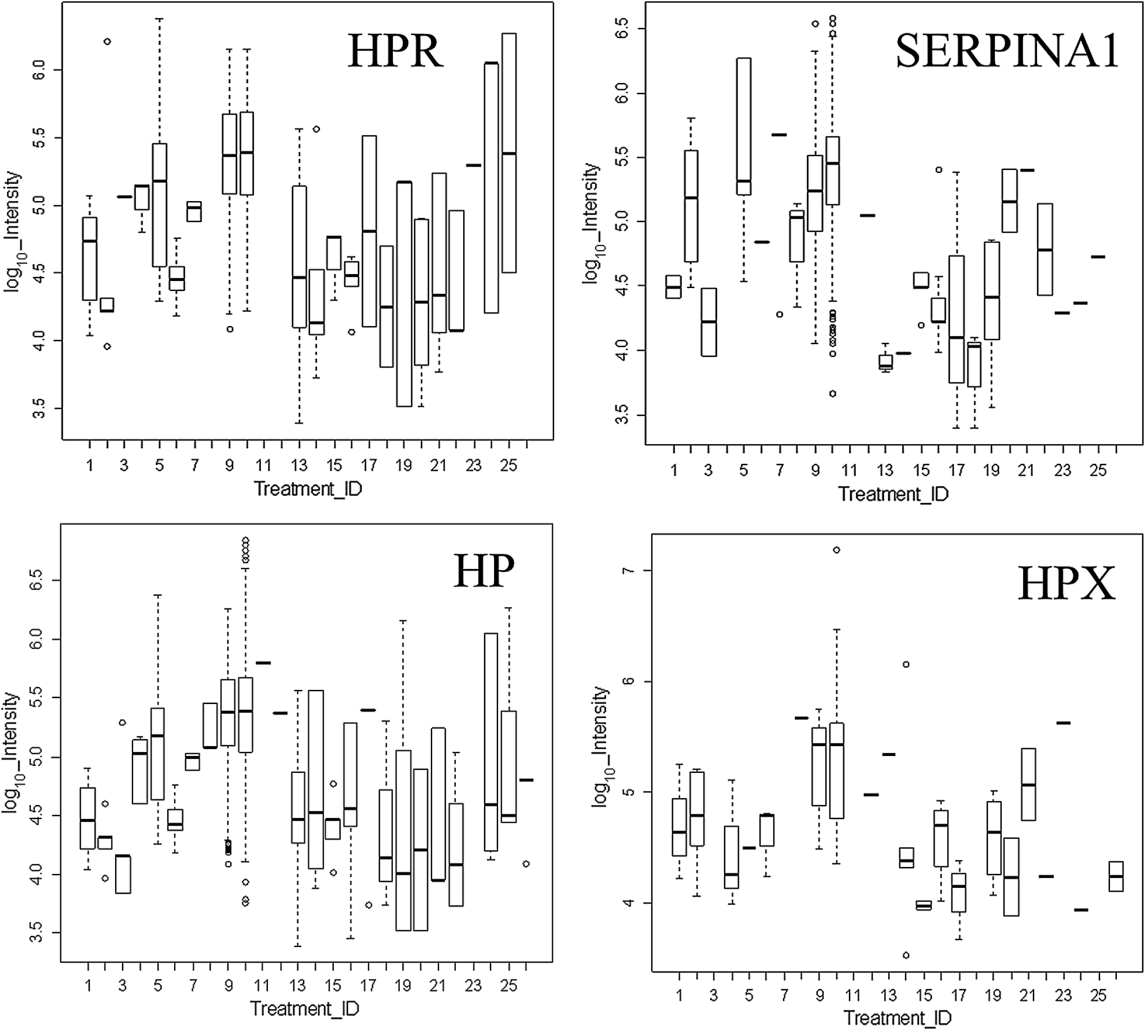
The variation in known plasma proteins across the clinical treatments. Treatment ID numbers: 1, Alzheimer normal; 2, Alzheimer normal control STYP; 3, AlzHeimer’s dementia; 4, Alzheimer’s dementia STYP; 5, Cancer breast; 6, Cancer breast_STYP; 7, Cancer_control; 8, Cancer control STYP; 9, Cancer ovarian; 10, Cancer ovarian_STYP; 11, Ice Cold; 12, Ice Cold STYP; 13, Heart attack Arterial; 14 Heart attack Arterial STYP; 15, Heart attack normal control, 16, Heart attack normal Control STYP; 17, Heart attack; 18, Heart attack STYP; 19, Multiple Sclerosis normal control; 20, Multiple Sclerosis normal control STYP; Multiple Sclerosis; 22, Multiple Sclerosis STYP, 23 Sepsis; 24, Sepsis STYP; 25, Sepsis normal control; 26, Sepsis normal control STYP. The ANOVA analysis of the proteins shown across treatments produced a significant F Statistic for means comparisons by Tukey–Kramer HSD test that showed significant differences between ovarian cancer or ovarian cancer STYP, versus the normal female control and/or breast cancer (see Additional file 1: Table S1, Additional file 2: Table S2 for Tukey–Kramer results for each protein shown). STYP: serine, threonine, tyrosine phosphorylation. Note that many proteins were not detected in the ice cold plasma

**Fig. 6:**
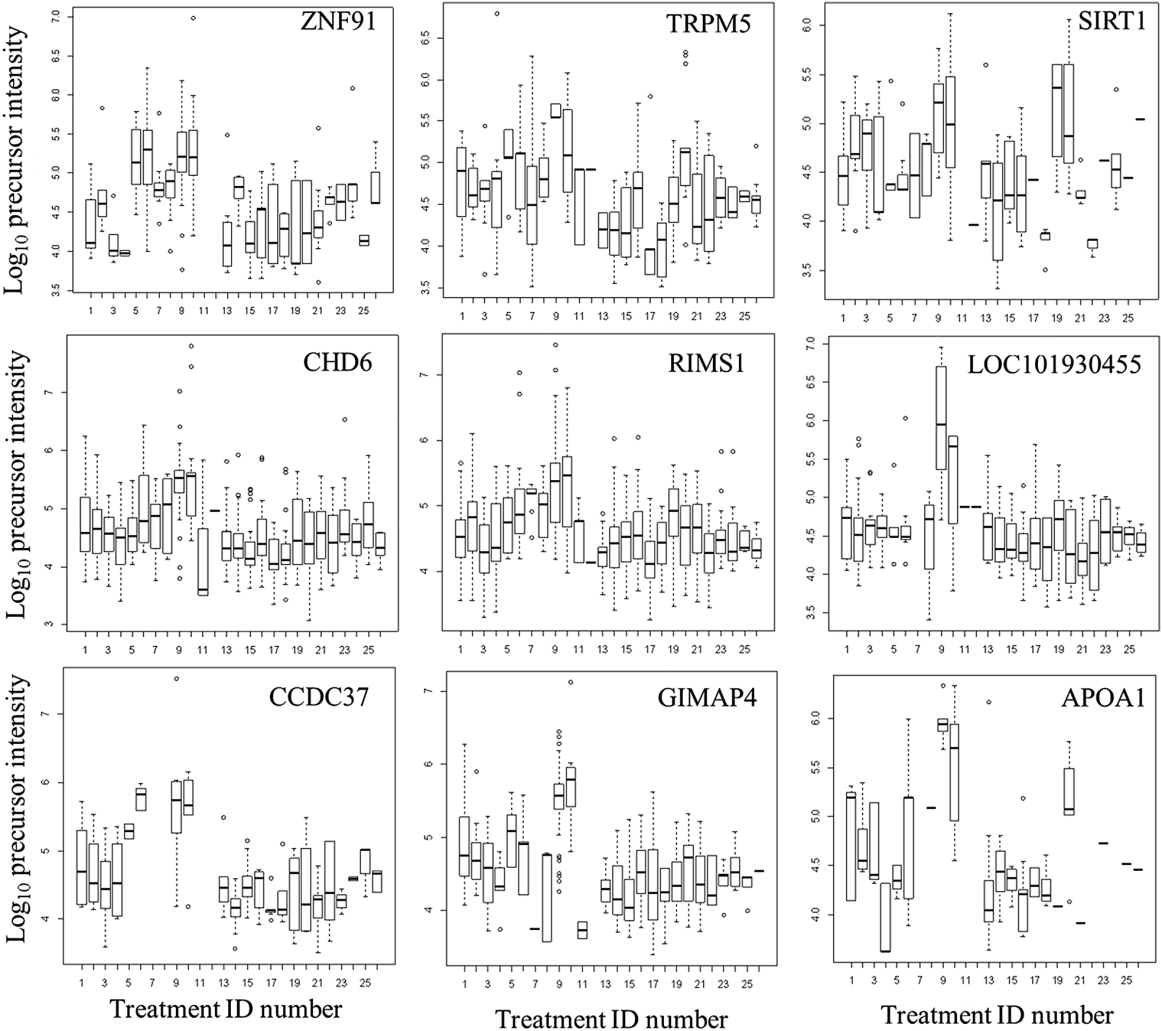
The variation in apparently cellular proteins in plasma across the clinical treatments. Treatment ID numbers: 1, Alzheimer normal; 2, Alzheimer normal control STYP; 3, Alzheimer’s dementia; 4, Alzheimer’s dementia STYP; 5, Cancer breast; 6, Cancer_breast STYP; 7, Cancer control; 8, Cancer control STYP; 9, Cancer ovarian; 10, Cancer ovarian STYP; 11, Ice Cold; 12, Ice Cold STYP; 13, Heart attack Arterial; 14 Heart attack Arterial_STYP; 15, Heart attack normal control, 16, Heart attack normal Control STYP; 17, Heart attack; 18, Heart attack STYP; 19, Multiple Sclerosis normal control; 20, Multiple Sclerosis normal control STYP; Multiple Sclerosis; 22, Multiple Sclerosis STYP, 23 Sepsis; 24, Sepsis STYP; 25, Sepsis normal control; 26, Sepsis normal control STYP. The ANOVA analysis of the proteins shown across treatments produced a significant F Statistic for means comparisons by Tukey–Kramer means comparison that showed a significant difference between ovarian cancer or ovarian cancer STYP (see Additional file 1: Table S1, Additional file 2: Table S2 for Tukey–Kramer results for each protein shown). STYP: serine, threonine, tyrosine phosphorylation. Note that many proteins were not detected in the ice cold plasma

Many proteins that showed greater observation frequency in ovarian cancer also showed significantly greater precursor intensity compared to breast cancer, the female normal controls, male and female EDTA plasma from other diseases and normals by ANOVA comparison. The mean precursor intensity values from gene symbols that varied by Chi Square (χ^2^ > 15) were analyzed by univariate ANOVA followed by the Tukey–Kramer Honestly Significant Difference (HSD) test in R [1, 23] (Table 3, Figs. 4, 5 and 6). For example, HPR showed precursor intensity quantile plots with  a linear and Gaussian distribution that ranged from E4 to more than E6 (Fig. 4). The common acute phase proteins HP, HPR, HPX, and SERPINA all showed significant increases with ovarian cancer (Fig. 5). Ovarian cancer showed a higher intensity of cellular proteins including Zinc Finger protein 91 (ZFN91), apparently extracellular protein LOC101930455 (XP_005275896 spidroin-1-like), Regulating Synaptic Membrane Exocytosis 1 (RIMS1), Transient Receptor Potential cation channel subfamily M member 5 (TRPM5), Helicase DNA Binding Protein 6 (CHD6), GTPase IMAP Family Member 4 (GIMAP4), and others by ANOVA followed by the Tukey–Kramer HSD test (Fig. 6). However, many proteins showed no difference between the ovarian versus the breast cancer clinical treatments such as APOA1 (Fig. 6).

**Table 3:** The analysis of mean peptide intensity per gene symbol for Haptoglobin related protein by ANOVA with Tukey–Kramer multiple means comparison

Treatment_ID	Mean	SD	SE (mean)	data:n	Tukey
1	4.62	0.35	0.08	20	bc
2	4.63	0.91	0.30	9	abc
3	5.07	NA	NA	1	cd
4	5.03	0.19	0.11	3	cd
5	5.12	0.58	0.09	46	ad
6	4.46	0.17	0.05	14	c
7	4.97	0.07	0.03	6	cd
9	5.34	0.42	0.02	687	d
10	5.35	0.41	0.01	951	d
13	4.51	0.64	0.14	21	c
14	4.45	0.65	0.17	14	c
15	4.63	0.23	0.09	7	abc
16	4.45	0.18	0.06	8	bc
17	4.81	0.81	0.40	4	cd
18	4.25	0.63	0.45	2	abc
19	4.62	0.86	0.35	6	abc
20	4.30	0.58	0.20	8	c
21	4.45	0.63	0.21	9	c
22	4.43	0.49	0.22	5	abc
23	5.30	0.00	0.00	2	cd
24	5.31	1.01	0.45	5	bd
25	5.38	1.02	0.51	4	bd

Response: log_10__Intensity

Sum Sq Df F value Pr(>F)

Peptide_Sequence 102.746 58 13.867 < 2.2e−16 ***

Treatment_ID 29.231 18 12.712 < 2.2e−16 ***

Peptide_Sequence:Treatment_ID  21.039   37   4.451 < 2.2e−16 ***

Residuals 219.478 1718

Treatment ID numbers: 1, Alzheimer normal; 2, Alzheimer normal control STYP; 3, Alzheimer’s dementia; 4, Alzheimer’s dementia STYP; 5, Cancer breast; 6, Cancer breast STYP; 7, Cancer control; 8, Cancer control STYP; 9, Cancer ovarian; 10, Cancer ovarian STYP; 11, Ice Cold; 12, Ice Cold STYP; 13, Heart attack Arterial; 14, Heart attack Arterial STYP; 15, Heart attack normal control; 16, Heart attack normal Control STYP; 17, Heart attack; 18, Heart attack STYP; 19, Multiple Sclerosis normal control; 20, Multiple Sclerosis normal control STYP; 21, Multiple Sclerosis; 22, Multiple Sclerosis STYP; 23, Sepsis; 24, Sepsis STYP; 25, Sepsis normal control; 26, Sepsis normal control STYP. STYP: serine, threonine, tyrosine phosphorylation. Note that many proteins were not detected in the ice cold plasma

## Discussion

Random and independent sampling of peptides from step-wise fractionation followed by LC–ESI–MS/MS is a time and manual labor intensive approach that is sensitive, direct, and rests on few assumptions [2, 56]. High signal to noise ratios of blood peptides is dependent on sample preparation to partition the sample into many selective sub-fractions to relieve competition and suppression of ionization and thus achieve sensitivity [24–26] but then requires large computing power to re-assemble, organize and analyze the sub-fractions together into samples within treatments for statistical analysis [21, 24–26, 56]. Here three independent lines of evidence, Chi Square analysis of observation frequency, ANOVA analysis of peptide intensity, together with previously established structural/functional relationships from STRING all agreed that there was significant differences in the peptides from specific proteins of ovarian cancer patients compared to controls. The previous careful study of pre-clinical variation over time, and under various storage and preservation conditions, seems to rule out pre-clinical variation as the most important source of variation between ovarian cancer and other disease and control treatments [2–4]. Together the results amount to a successful proof of principal for the application of random and independent sampling of plasma from ovarian cancer versus multiple clinical treatments by LC–ESI–MS/MS to identify and quantify proteins and peptides that show variation between sample populations.

### Pre-analytical variation

Collecting blood plasma samples directly onto ice might prevent the secretion of enzymes or proteins from blood cells, and prevent the degradation of proteins by proteases ex vivo. The effect of ex vivo proteolysis on the endogenous peptides of blood samples can be prevented by acid quench, protease inhibitors, freeze drying or ice to preserve the sample [1, 2, 4, 5]. EDTA plasma from blood collected on ice was stable when freeze dried with low peptide frequency and intensity but liquid plasma slowly degrades at room temperature [2, 4, 5]. Blood fluid contains a net weak tryptic activity [57] that may cleave endogenous peptides in vivo (peptidome) and endogenous proteolytic activities generate high levels of some of these same peptides ex vivo (degradome) [58, 59] where these two pools show some overlap [2]. The frequency and/or intensity of peptide observations increased in samples incubated at room temperature compared to ice cold samples that shared some peptides and proteins [1–3, 5, 24]. The increased frequency and average precursor intensity values of cellular proteins across the clinical samples compared to the ice cold controls indicates the some of the peptides and or proteins observed were released from cells, or degraded by proteases released or activated, ex vivo. There was apparently statistically significant variation in the cleavage of endogenous peptides from cellular proteins across the different disease and normal treatments, female samples and ice cold controls.

### Chi Square analysis of ovarian cancer versus female normal

Specific endogenous tryptic peptides, were detected from ovarian cancer versus the corresponding normal female or the other diseases and controls. The large differences in observation frequency support the existence of disease-specific peptides in the blood plasma of ovarian cancer patients. The results here with Haptoglobin (HP) in Ovarian Cancer agree with previous results [39]. Large increases in the frequency and intensity of Haptoglobin Related Protein (HPR), alpha antitrypsin (SERPINA1), Hemopexin (HPX) or other proteins were observed, but the greater representation of these common, acute-phase response proteins is not likely to be highly specific to one disease [38]. Many of the proteins that were significantly increased in disease, compared to the 6 sets of controls, included amyloids, complements, haptoglobin, IgG chains, IITI, anti-trypsin, alpha 2 macroglobulin, fibrinogens, hemopexin, apolipoproteins that are elevated in more than one disease [38]. However, specific phosphorylations or other post translational modifications of acute phase or other common blood proteins might provide some greater utility than increases in these proteins alone [5, 60–63]. Many of the proteins that varied in ovarian cancer were previously shown to play a role in cancer biology, or were previously established tumor diagnostic or prognostic markers and several have previously been detected in the plasma of cancer: Coagulation factor XIII has been suggested to be a biomarker for screening colorectal cancer [9]; P4HA1 is a prolyl 4-hydroxylase that may be a prognostic marker for glioma [64]; Glipican has been localized to exosomes and previously implicated as a biomarker of cancer [42]; Laminin B2 promotes non-small cell lung cancer [65]; CSR1 is a tumor suppressor gene that activates CPSF3 preventing the interaction of XIAP with caspase [66]; MORN3 is a testes-cancer antigen that recruits the Sirtuin deacetylase that modifies P53 [67]; SIRT1 (Sirtuin) is a histone deacetylase that may regulate tumor formation [68]; Cyclin 1-like (CCN12) plays a role in cell cycle progression and proliferation [69]; NMI is an N-MYC and STAT interactor shown to increase in protein expression with tumor grade and plays a role in cell cycle progression [70]; Increased ITGB1 integrin beta 1 has been shown to be associated with some, but not all, solid cancers [71]; A gene expression array identified NEFM as indicative of the risk of prostate cancer [72]; PLEC1 was shown to promote esophageal cancer cell progression by maintaining the expression of SNAIL [73]; SRGN was show to be expressed in the exosomes of adenocarcinoma by LC–ESI–MS/MS [74]; DHCR reduces cholesterol, may play a role in cancer [75] and selective and potent inhibitors of DHCR have been developed [76]; SMC5 complexes with MMS21 that acts as an E3 ligase required to avoid gross chromosomal rearrangements [77]; Semaphorins such as SEMA6B were strongly down regulated in breast cancer [78]; Lysyl oxidase-like 3 was required for melanoma cell survival [79]; Seizure related 6 homolog (SEZ6L2) showed increased gene expression in primary lung cancer by RT-PCR and Western blot [80].

### Pathway and gene ontology analysis by the STRING algorithm

The set of gene symbols that were significant from Chi Square analysis of the peptide frequency counts were independently confirmed by STRING analysis. The network analysis by STRING indicated that the peptides and proteins detected were not merely a random selection of the proteins from the human genome but seemed to show statistically significant protein–protein interactions, and showed significant enrichment of cellular components, biological processes, and molecular functions associated with the biology of cancer. The significant results from STRING analysis seemed to indicate that at least some of the differences observed could not have resulted from random sampling error between ovarian cancer and the female normal controls. The previously established structural or functional relationships observed among the ovarian cancer specific gene symbols filtered by χ^2^ were consistent with the detection of bone fide variation specific to ovarian cancer. The STRING results apparently indicate that specific protein complexes are released into the circulation of ovarian cancer patients [40].

### Ovarian cancer specific variation by ANOVA

After testing the discrete frequency data using the computationally extensive Chi Square (χ^2^) test, the significant protein gene symbols were then analyzed by computationally intensive ANOVA of the continuous and normally distributed (Gaussian) log_10_ intensity values [22, 23, 35]. A potential role has been suggested for ZNF91 in some cancer pathogenesis [81, 82] and zinc finger proteins may play a role attenuating the cellular effects of viral genes [83] that may account for some 15% of cancer [84]. The large zinc finger superfamily that may bind RNA and DNA have been detected in human blood by partition chromatography, organic extraction of endogenous peptides and Western blot [25, 26, 30]. Regulation of the chromatin remodeling enzyme CHD6 was observed in the molecular analysis of urothelial cancer cell lines [85]. A novel translocation of LMBRD1-CHD6 (6;20)(q13;q12) was observed in acute myeloid leukemia [86]. Dis-regulation of CHD6 was also observed in models of colorectal cancer [87]. Sirtuin 1 (SIRT1) may promote cellular proliferation, migration and invasion in epithelial ovarian cancer [88] and inhibits p53-dependent apoptosis in human melanoma cells [89]. Hemopexin is expressed in a model of hepatocellular carcinoma from hepatitis B in woodchucks [90]. In contrast, there is no previous study of LOC102723511, (adhesive plaque matrix protein-like) that remains a hypothetical protein. Similarly, the glycine rich unknown protein XP_005275896 that is encoded by LOC101930455 may show some cryptic sequence homology to bacterial proteins and general features consistent with extracellular structural proteins that might be important for biochemical marker development [62]. In general, many of the proteins that showed greater frequency and/or intensity in ovarian cancer from plasma peptides were consistent with the previously established role of the proteins in cancer or tumor biology.

### Ovarian cancer EDTA plasma peptides and proteins

It is not clear if the observed variation results from greater expression of the specific proteins, expression of proteases that target the observed proteins, greater susceptibility to endoproteolytic attack, greater resistance to exopeptidase activity, or the combinations, as the source of variation between proteins and sample treatments. It should be possible to specifically compare and confirm the levels of disease specific peptides and parent proteins by automatic targeted proteomics [4] after extraction of peptides in one step [30] or after collection of the intact protein chains over the best partition chromatography resin [26] followed by tryptic digestion and analysis. For example, C4B peptides discovered by random and independent sampling were shown to be a marker of sample degradation by automatic targeted assays [2–4]. Automatic targeted analysis of peptides from independent analysis provided relative quantification to rapidly confirm the potential utility of C4B peptide as a marker of sample degradation [4]. There is strong evidence that the action of disease-specific tryptic endoproteinase activity cleaves specific peptides in blood fluids that may sensitivity reflect changes in the corresponding parent proteins [1]. We cannot rule out that at least some of the endogenous peptides detected more specifically in ovarian cancer may reflect an increased concentration of the parent protein [38]. Attempts to analyze the proteins of blood by depletion and tryptic digestion first, followed by separation of peptides over strong cation exchange and C18 cannot be used to focus on one protein in a targeted manner [91]. In contrast, the separation of the proteins first by partition chromatography followed by tryptic digestion of the enriched fraction and C18 separation of peptides may permit the efficient, and automated, targeted assay of specific proteins without the use of immunological reagents [26]. Traditional partition chromatography using quaternary amine, propyl sulfate, concanavalin A, heparin or DEAE resin followed by trypsin digestion and LC–ESI–MS/MS robustly identify at least 4396 blood proteins by X!TANDEM using disposable preparative micro chromatography resins followed by LC-ESI-MS/MS [25, 26]. Thus one step organic extraction [27], and/or the partition chromatography of the parent proteins followed by tryptic digestion [25, 26], may be used to automatically confirm the peptides and proteins and provide relative quantification by ANOVA [35]. Subsequently, the best performing peptides and proteins may be absolutely quantified by external or internal isotopic standards [92].

## Conclusion

The step wise organic extraction of peptides [24] provided for the enrichment of endogenous tryptic peptides with high signal to noise for random sampling [4] across disease and control (normal) treatments. A large amount of proteomic data from multiple diseases, controls and institutions may be stored, related and statistically analyzed in 64 bit SQL Server/R. The random and independent sampling of plasma endogenous tryptic peptides by LC-ESI-MS/MS identified many new blood proteins that were previously associated with the biology of cancer or that have been shown to be biomarkers of solid tumors by genetic or biochemical methods. The striking level of agreement between the results of random and independent sampling of plasma by mass spectrometry with those from cancer tissues and cells seems to indicate that clinical discovery of plasma by LC–ESI–MS/MS will be a powerful tool if it can be applied at a larger scale. A larger scale of extraction, and larger C18 preparative bed volume, would be required to automate the discovery and confirmation process for clinical applications by a modification of the existing method [24] to create a highly concentrated sample sufficient to fill and saturate the surface of an auto-sampling vial. Previous C4B peptides that were discovered as markers of sample degradation by random and independent sampling of tryptic peptides and were subsequently confirmed by automatic targeted analysis from independent samples [2–4] that strongly indicate a similar work flow could be applied to disease versus normal samples. 

## Additional files

**Additional file 1: Table S1** The number of successful LC-ESI-MS/MS experiments that resulted in successful correlations to peptides from the various disease and normal treatments.

**Additional file 2: Table S2** Average Chi Square value per gene symbol for ovarian cancer versus normal female plasma.
