# Supplementary material for: The plasma peptides of ovarian cancer
Source: Clin Proteomics. 2018 Dec 21;15:41. doi: 10.1186/s12014-018-9215-z (PMC6302491; doi:10.1186/s12014-018-9215-z)
Supplement: Supplementary file 1 — Additional file 1: Table S1 The number of successful LC-ESI-MS/MS experiments that resulted in successful correlations to peptides from the various disease and normal treatments. [file 12014_2018_9215_MOESM1_ESM.docx]

Additional file 1: Table S1. The number of successful LC-ESI-MS/MS experiments that resulted in successful correlations to peptides from the various disease and normal treatments.
